# A squeezed light source operated under high vacuum

**DOI:** 10.1038/srep18052

**Published:** 2015-12-14

**Authors:** Andrew R. Wade, Georgia L. Mansell, Sheon S. Y. Chua, Robert L. Ward, Bram J. J. Slagmolen, Daniel A. Shaddock, David E. McClelland

**Affiliations:** 1 Centre for Gravitational Physics, Department of Quantum Science, The Australian National University, Canberra, 2601, Australia; 2 Now at Laboratoire Kastler Brossel, UPMC-Sorbonne Universités, CNRS, ENS-PSL Research University, Collége de France, Paris, France

## Abstract

Non-classical squeezed states of light are becoming increasingly important to a range of metrology and other quantum optics applications in cryptography, quantum computation and biophysics. Applications such as improving the sensitivity of advanced gravitational wave detectors and the development of space-based metrology and quantum networks will require robust deployable vacuum-compatible sources. To date non-linear photonics devices operated under high vacuum have been simple single pass systems, testing harmonic generation and the production of classically correlated photon pairs for space-based applications. Here we demonstrate the production under high-vacuum conditions of non-classical squeezed light with an observed 8.6 dB of quantum noise reduction down to 10 Hz. Demonstration of a resonant non-linear optical device, for the generation of squeezed light under vacuum, paves the way to fully exploit the advantages of in-vacuum operations, adapting this technology for deployment into new extreme environments.

Non-classical light states are envisioned to have many practical uses, including quantum networks^1^ and cryptography^2^, quantum computation^3^, biophysics^4^, and gravitational wave (GW) detection^5^,^6^,^7^. Applications such as improving the sensitivity of advanced gravitational wave detectors^8^ and the development of space-based quantum networks^9^ will require field-deployable, vacuum-compatible non-classical light sources. To date non-linear photonics devices operated under high vacuum have been simple single pass systems, testing harmonic generation^10^,^11^ and the production of classically correlated photon pairs^12^ for space-based applications. Here we demonstrate the production under high-vacuum conditions of non-classical squeezed light with an observed 8.6 dB of quantum noise reduction down to 10 Hz. This performance is comparable to non-linear photonics devices previously demonstrated under in-air laboratory conditions^5^,^13^,^14^,^15^,^16^. Demonstration of a squeezed light source under vacuum paves the way to fully exploit the advantages of in-vacuum operations, such as the production of squeezed light in space, or in the case of gravitational wave detectors, advanced acoustic and seismic isolation.

When laser interferometer type gravitational wave detectors, such as the Advanced Laser Interferometer Gravitational Wave Observatory (LIGO)^17^, Advanced Virgo^18^, and KAGRA^19^, reach their design sensitivity of roughly 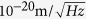 at 100 Hz, they will be limited by quantum noise across much of the detection band. This quantum noise arises from the quantum mechanical fluctuations of the electromagnetic field used to sense displacement and is not related to any quantum behaviour of the mechanical test masses^20^. Furthermore, laser interferometer gravitational wave detectors typically operate on (or very near) a dark fringe, where the laser injected to the input port is reflected back to the source. This has the primary advantage of rejecting most of the classical laser noise, and it also rejects the quantum noise in the laser field. A similar reflection occurs at the output, or readout, port of the interferometer. When there is no laser source present at the output port, the vacuum state of the electromagnetic field enters instead and is reflected from the interferometer; the quantum mechanical fluctuations in this vacuum state are the origin of the quantum noise^21^.

Reductions in quantum noise can be realised by replacing the vacuum field, that would otherwise enter the readout port, with a carefully engineered non-classical light state, a so-called squeezed vacuum state^21^. In an ordinary vacuum state, the vacuum fluctuations at different frequencies are uncorrelated, leading to quantum noise in the measurement process. In a squeezed vacuum state, the vacuum fluctuations at Fourier frequencies 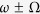 are correlated, where *ω* is the central squeezing frequency. These correlations can yield a reduced quantum noise.

For frequency independent squeezing (the squeeze factor and angle do not change with Ω) such as that demonstrated here, the changes in the quantum noise performance of an interferometer induced by squeezing are equivalent to changing the input laser power. Increasing the laser power to improve the shot noise limited signal-to-noise ratio confronts challenging limits in absorption induced thermal distortions of mirrors^22^, parametric instabilities that limit instrument stability^23^, and scattered light^24^. Injection of squeezed vacuum states of light offers an alternative strategy for reducing quantum shot noise, provided a source of squeezed vacuum can be realised that is both stable and contributes minimal additional noise within the detector’s 10 Hz–10 kHz ‘audio’ band; this is the important frequency band for ground-based gravitational wave detection. Squeezed light injection has been demonstrated in the GEO600^5^,^25^ and the LIGO GW detectors^15^.

The highest-performance squeezed light sources are optical parametric oscillators (OPO)^26^. These are optical cavities with an embedded second-order nonlinear medium. The cavity resonant frequency is the central squeezing frequency; when the nonlinear medium is pumped with the second harmonic of this frequency, the OPO is degenerate and the parametrically down-converted signal and idler fields are equally distant from the central frequency. For the case of squeezed vacuum, these signal and idler fields are the correlated upper and lower noise sidebands due to vacuum fluctuations.

The integration of a squeezed light source into the output port of a GW detector must preserve squeezing without introducing new noise pathways. Scattered light^26^,^27^, optical losses, and squeezed quadrature angle fluctuations^28^,^29^ due to length noise and alignment jitter are all obstacles to realising high levels of quantum noise suppression. We address these design considerations with a doubly-resonant travelling wave OPO^16^ made nearly entirely of high quality fused silica glass designed for ultra-high vacuum operation (see Fig. 1), with a non-linear crystal made of periodically poled potassium titanyl phosphate (PPKTP). The OPO has been designed to be similar in dimensions and materials (with exception of prism epoxy bonding) to the Advanced LIGO output mode cleaner cavity^17^ to take advantage of existing seismic and acoustic isolation platforms.

The near monolithic glass design is expected to provide low length noise and long term alignment stability, reducing quadrature and angular fluctuations, while the travelling-wave topology reduces the impact of scattered light^16^. Placing the squeezed light source in the main vacuum envelope allows significant acoustic and seismic isolation, which further reduces the impact of scattered light. This reduction of scattered light, in turn, reduces the required amount of optical isolation (usually accomplished via Faraday isolators) and the concomitant optical loss.

The permanently fixed cavity mirrors must be precisely and accurately positioned and angled. The OPO cavity was constructed by optically contacting mirror-glass block assemblies to a highly polished glass breadboard. Optical contacting offers a strong direct intermolecular bond with pitch guaranteed to the flatness of the breadboard and squareness of optical blocks (±0.18” and ±30” respectively), an important advantage for keeping the optical axis well centred on all optics. Solution assisted contacting is employed to mediate the speed of the contact as the blocks are moved into place. A precision cut aluminium alignment mask was used to guide the alignment. A bowtie resonator was formed with a 6° angle of incidence and a total cavity roundtrip length of 345 mm. A 30 *μm* (21.2 *μm*) fundamental 1064 nm (pump 532 nm) waist is formed between the two −50 mm radius of curvature concave mirrors. A visible 532 nm beam was used to establish the cavity eigenmode as micrometers were used to fine-adjust the mirror yaw as mirror-breadboard contact finally set. The goodness of the contact is integral to the mechanical stability of the device. Considering that the non-adjustable mirrors in the cavity must be aligned within a limited time window, this is an important demonstration of the viability of optical contacting for a four mirror bowtie cavity, and thus for further OPOs for use in extreme environments and/or with exacting mechanical requirements.

To maintain the phase matching condition between the pump and fundamental light the non-linear crystal must be precisely temperature controlled while under vacuum. Temperature of the PPKTP crystal was controlled via a standard proportional-integral-derivative controller feeding back to an ‘oven’ constructed from vacuum compatible materials while maintaining adequate thermal contact. In air, close contact between the crystal and surrounding copper is enough to control the crystal temperature, with sufficient thermal conduction primarily provided through a residual air layer between the irregular surfaces of the crystal and the copper (neither of which are super polished). For vacuum operation, rather than using a thermally conductive paste, a layer of indium foil was sandwiched between each element in the oven to replace the conduction provided by the residual air at atmospheric pressure. Thermally conductive pastes have unproven vacuum compatibility with the potential to outgas contaminants that may damage low-loss optical coatings in close proximity.

For dual resonance of the pump and fundamental light, the dispersion of the optical elements in the resonator, including the non-linear crystal, must be compensated. This is accomplished by wedging the non-linear crystal (1.15°); translating the crystal across the beam changes the propagation distance in the crystal material, which when combined with the dispersion in the crystal can be used to ensure dual resonance of the pump (532 nm) and fundamental squeezed fields (1064 nm). The OPO is constructed in standard laboratory conditions, which includes air at 1 atmosphere. The dispersive shift between pump and fundamental wavelengths in air 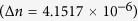
30 corresponds to roughly 1.3 free spectral ranges of the cavity for the fundamental 1064 nm field; this change in dispersion must be compensated when moving to a vacuum environment. Fine adjustments to the cavity dual resonance condition can also be realised by fine tuning the crystal temperature; this also changes the phase matching.

Figure 2 illustrates the shift of the fundamental field away from the ideal co-resonance when the cavity is held to resonance in 532 nm while the cavity is placed under vacuum. Overlaid is the phase matching window of the PPKTP crystal along with the expected squeezing level. A double resonance condition under vacuum may be achieved by anticipating the change in dispersion, and setting the crystal position to achieve simultaneous phase matching and co-resonance once the air is evacuated. This reduces the available squeezing level while at atmospheric pressure.

The experimental setup is shown in Fig. 3. Pump (532 nm) light is generated and injected into the doubly resonant glass OPO cavity situated in a vacuum tank maintained at 1 × 10^−6 ^mBar. Out coupled squeezed light was directed out of the vacuum and measured in a balanced homodyne detector arrangement.

Results from in vacuum operation of the OPO are shown in Fig. 4. Excluding points affected by coupling from supply AC, the achieved squeezing is 8.6 ± 0.9 dB of noise reduction below the shot noise level with anti-squeezing in the orthogonal quadrature of 15.9 ± 0.7 dB. Operation of the OPO was demonstrated for periods of over an hour with the device operational after weeks in vacuum; no performance degradation has been observed.

We have operated an optical parametric oscillator under high-vacuum conditions producing squeezed optical vacuum and a reduction of the quantum noise level by 8.6 dB in the audio band; this is comparable to similar in-air audio-band squeezing experiments. We have identified a number of issues for non-linear photonics devices including the need to account for dispersion of air when moving to vacuum. The demonstration of an all glass monolithic OPO cavity operating in vacuum opens the path for development of similar designs to be implemented within the seismically isolated vacuum environments of advanced gravitational wave detectors. Installation of such a vacuum compatible squeezed light source, especially if paired with a filter cavity to produce frequency dependent squeezing^31^, has the potential to significantly improve the quantum limited sensitivity in future gravitational wave detectors.

## Methods

The cavity length is locked to resonance for the pump light using a 70 MHz RF sideband Pound-Drever-Hall locking scheme in reflection (PD-PDH), with the actuation provided by PZTs to which the curved cavity mirrors are bonded. The vacuum field is de-amplified in quadrature with the pump light, generating squeezed and anti squeezed quadrature vacuum states. The phase of the pump must therefore be controlled relative to the homodyne LO field to select a chosen quadrature (squeezing or anti-squeezing). A modified coherent locking scheme^16^,^32^ was implemented by means of an auxiliary (1064 nm) laser. This was frequency offset by 29.8 MHz from the OPO fundamental field via a frequency doubled beat note (PD1) locked at 59.6 MHz offset from the OPO pump field. The phase relationship between the 29.8 MHz subcarrier and the pump is therefore fixed and they are co-propagated into the OPO cavity sharing a common path. A phase dependant error signal is derived at the balanced homodyne detector from the transmitted 29.8 MHz coherent control sidebands and the local oscillator. Because of the beat note lock with the pump light, this error signal tracks the phase of the pump light, allowing the locking of the homodyne quadrature of detection to the squeezed quadrature. These 1064 nm offset probe sidebands are far enough away from the fundamental field and enough out of the line-width of the cavity to not contaminate the vacuum squeezed field or deplete the available pump.

The power present at the input of the OPO cavity was set to 105 mW; this provides a classical non-linear gain of an injected seed field of approximately 16, and corresponds to approximately 75% of threshold. The input coupler power reflectivity of 0.845 (0.70) in the fundamental 1064 nm (pump 532 nm) field gives a cavity finesse of 37 (17.6). With a free spectral range of 849 MHz, that includes the crystal refractive index, gives a cavity line-width of 23 MHz and 48.3 MHz for the fundamental 1064 nm and pump 532 nm fields respectively. The 29.8 MHz coherent locking offset was close enough to the cavity line width to achieve a sufficient error signal.

The balanced homodyne measurement of the vacuum state is made with 1 mW of local oscillator (LO) light present on each photodetector. This field was mode cleaned and passed through a Faraday isolator to maximise the spatial quality of the available coherent light and to minimise potential backscatter. The homodyne setup provided 70 dB common mode rejection for LO noise. Considerable effort was invested to identify and mitigate sources of scattered light. Further environmental isolation was achieved by enclosing the homodyne detector and associated optics within a heavy aluminium box with 20 mm thick walls.

**Figure 4:**
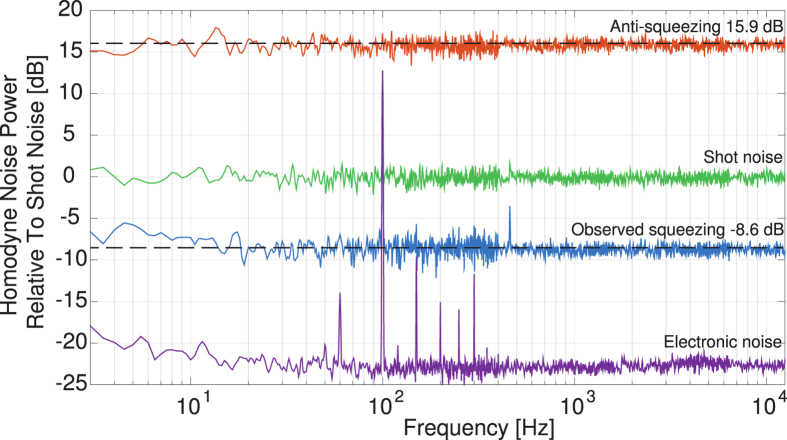
Noise power level relative to shot noise level. Shown are the quantum noise due to ordinary vacuum (Shot noise, green trace), the noise in the un-squeezed quadrature (anti-squeezing, orange trace), the noise in the squeezed quadrature (observed squeezing, blue trace), and the electronics baseline noise level (violet). See methods for estimate loss in the squeezing path of 8.9%. The noise spectra are normalised relative to the shot noise level but are not corrected for the electronic noise. Noise spikes at integer multiples of the 50 Hz power mains supply remain with a 460 Hz peak in all three measurement that is of an unknown origin.

Traces displayed in Fig. 4 were normalised to the shot noise level of a pure vacuum field and have a clearance above the electronic (dark) noise floor of over 20 dB. The resulting quantum noise suppression below shot noise value is limited by both the losses and phase noise between the OPO source and homodyne detector. Total loss is estimated to be 8.9 ± 0.1% which includes a 97.9 ± 0.1% OPO escape efficiency, 97.5 ± 0.5% propagation efficiency, a photodetector quantum efficiency (*η*_PD_) of 98 ± 1% and an homodyne mode mismatch contributing 97.4 ± 0.5%. This is estimated to have reduced the inferred initial squeezing level of 16.3 ± 0.7 dB to 9.50 ± 0.3 dB. Estimated phase noise was 21 ± 1 mrad rms based on measurements of squeezing as a function of tuned non-linear gain. Contributions of phase noise are estimated to have reduced the squeezing level further to 8.9 ± 0.3 dB by projecting a portion of the anti squeezed quadrature onto the squeezed quadrature measurement.

Improvements of the mode matching to 99.5% visibility should be expected to improve squeezing levels to 9 dB. With further improvements to phase noise to 11 mrad rms and propagation efficiency to 99%, measured squeezing may be improved to 10 dB.

## Additional Information

**How to cite this article**: Wade, A. R. *et al.* A squeezed light source operated under high vacuum. *Sci. Rep.* **5**, 18052; doi: 10.1038/srep18052 (2015).

## Figures and Tables

**Figure 1:**
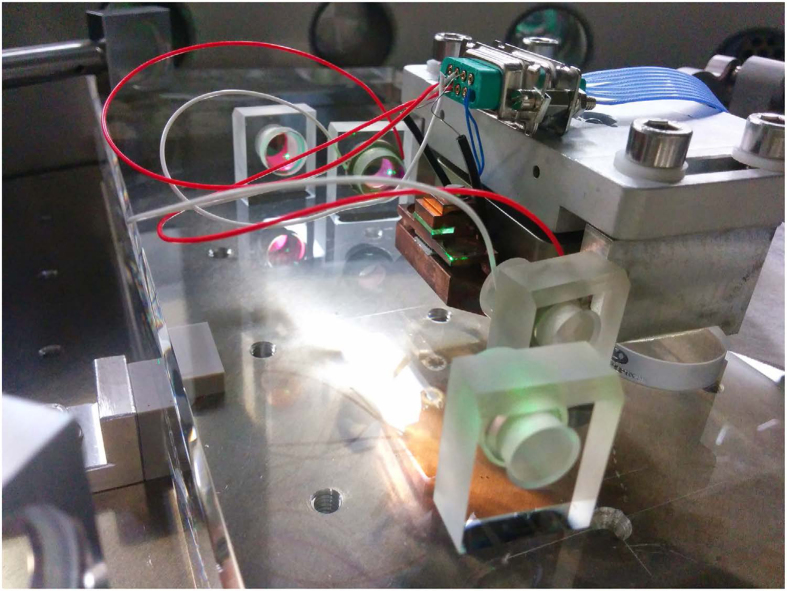
OPO cavity formed from blocks optically contacted to a glass breadboard (all fused silica). Half-inch mirrors are bonded to silica blocks at 15 mm height to form a traveling wave bow tie cavity with angle of incidence on the curved mirrors of 6° and a round trip length of 0.345 m. Two −50 mm concave mirrors focus pump (532 nm) light to waist of 21 *μm* with corresponding fundamental waist of 30 *μm* within a temperature controlled wedged PPKTP crystal. A Newport 9071-V stage is used to align and translate the crystal through the intra-cavity beam.

**Figure 2:**
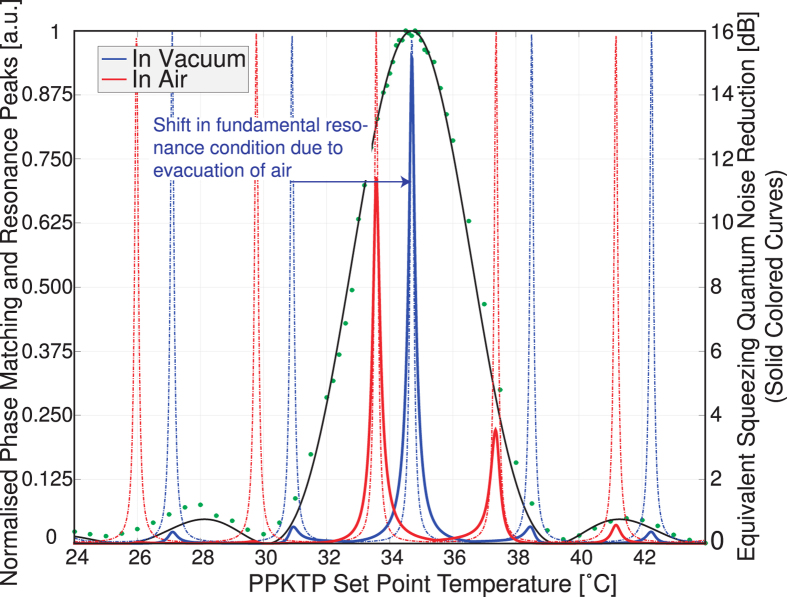
Phase matching data (circles) and theoretical curve (solid black line), with theoretical curves for the fundamental field resonance when tuned for air (dashed red) and vacuum (dashed blue), and available squeezing (solid red and blue). All curves plotted as a function of non-linear crystal temperature. For all curves, the pump field is considered to be held on resonance in the cavity.

**Figure 3:**
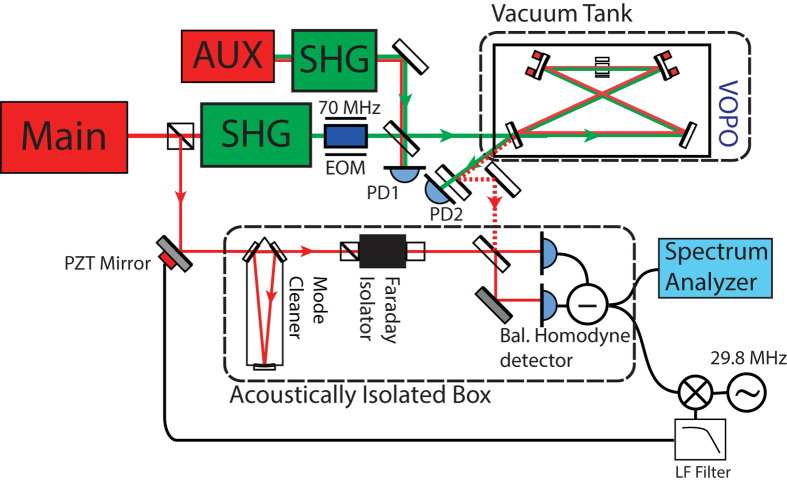
Schematic of experimental setup. The central squeezing frequency is that of the main 1064 nm laser (Main). The in-vacuum OPO (VOPO) is pumped by light from the main laser which has been frequency-doubled in external second harmonic generator (SHG). The OPO cavity is kept on resonance with the pump light via a Pound-Drever-Hall locking scheme, using 70 MHz sidebands applied with an electro-optic phase modulator (EOM) and the beam reflected from the cavity detected at PD2. To control the phase of the pump relative to the homodyne local-oscillator (LO), a modified coherent locking scheme^16^,^32^ was implemented by means of an auxiliary (1064 nm) laser (AUX), frequency shifted from the main laser by 29.8 MHz, that co-propagates with the squeezed light. This auxiliary laser is beat with the main laser at PD1 to maintain the frequency difference. The squeezed light signal was extracted by coupling out of the vacuum tank enclosure via a low loss vacuum window, stripping off pump (532 nm) light and performing a balanced homodyne detection. An error signal to control the relative phase of the local oscillator to the squeeze angle of the OPO is generated by mixing down the beatnote between the auxiliary laser coherent control sidebands and the LO; the LO path length is adjusted with a PZT-endowed mirror.
